# Pylorus-preserving pancreatoduodenectomy preserving the right gastroepiploic vessels following proximal gastrectomy: report of two cases

**DOI:** 10.1186/s40792-019-0599-4

**Published:** 2019-03-12

**Authors:** Teijiro Hirashita, Yukio Iwashita, Hiroaki Nakanuma, Kazuhiro Tada, Kunihiro Saga, Takashi Masuda, Yuichi Endo, Masayuki Ohta, Toshifumi Matsumoto, Masafumi Inomata

**Affiliations:** 1 Department of Gastroenterological and Pediatric Surgery, Oita University Faculty of Medicine, 1-1 Hasama-machi, Yufu, Oita 879-5593 Japan; 2 Department of Surgery, National Hospital Organization Beppu Medical Center, 1473 Uchikamado, Beppu, Oita 874-0011 Japan

**Keywords:** Pancreatoduodenectomy, Proximal gastrectomy, Right gastroepiploic vessel

## Abstract

**Background:**

Blood flow of the remnant stomach is supplied via the right gastric and right gastroepiploic vessels after proximal gastrectomy (PG). Whether the remnant stomach can be safely preserved in patients who undergo pylorus-preserving pancreatoduodenectomy (PPPD) after PG remains unclear. We herein report two cases in which the remnant stomach was safely preserved by performing PPPD.

**Case presentation:**

The first patient, a 76-year-old man, was diagnosed with cancer of the common bile duct and underwent PPPD 2 years after PG for gastric cancer. The remnant stomach and right gastroepiploic vessels were safely preserved. The second patient, a 56-year-old man with a history of PG for gastric cancer 20 years previously, was diagnosed with cancer of the common bile duct and underwent PPPD. We could safely preserve the remnant stomach and right gastroepiploic vessels.

**Conclusion:**

The remnant stomach could be preserved in performing PPPD following PG by preserving the right gastroepiploic vessels.

## Background

Blood supply is important for preserving the remnant stomach after gastrectomy when pancreatectomy is performed [1]. We sometimes encounter patients who need to undergo distal pancreatectomy after distal gastrectomy, and some reports have discussed whether the remnant stomach can be preserved or not [2, 3]. Blood flow for the remnant stomach is supplied via the right gastric and gastroepiploic vessels after proximal gastrectomy (PG). To preserve the remnant stomach in patients who undergo pylorus-preserving pancreatoduodenectomy (PPPD) after PG, the right gastric or gastroepiploic vessels should be preserved or reconstructed. There are only a few reports on performing PPPD after PG with preservation or reconstruction of gastric vessels [4, 5]. We herein report two cases in which the remnant stomach was safely preserved by performing PPPD after PG.

## Case presentation

### Case 1

**Fig. 1:**
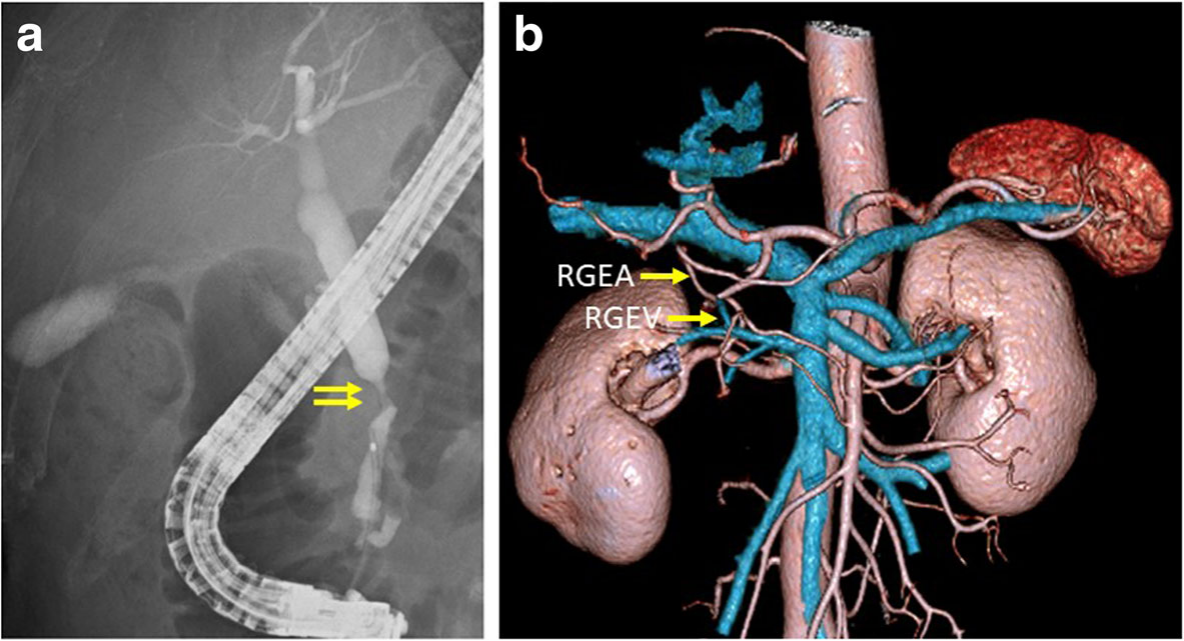
**a** ERC showed stenosis of the common bile duct with a diameter of 15 mm. **b** CT reconstructing blood vessels

**Fig. 2:**
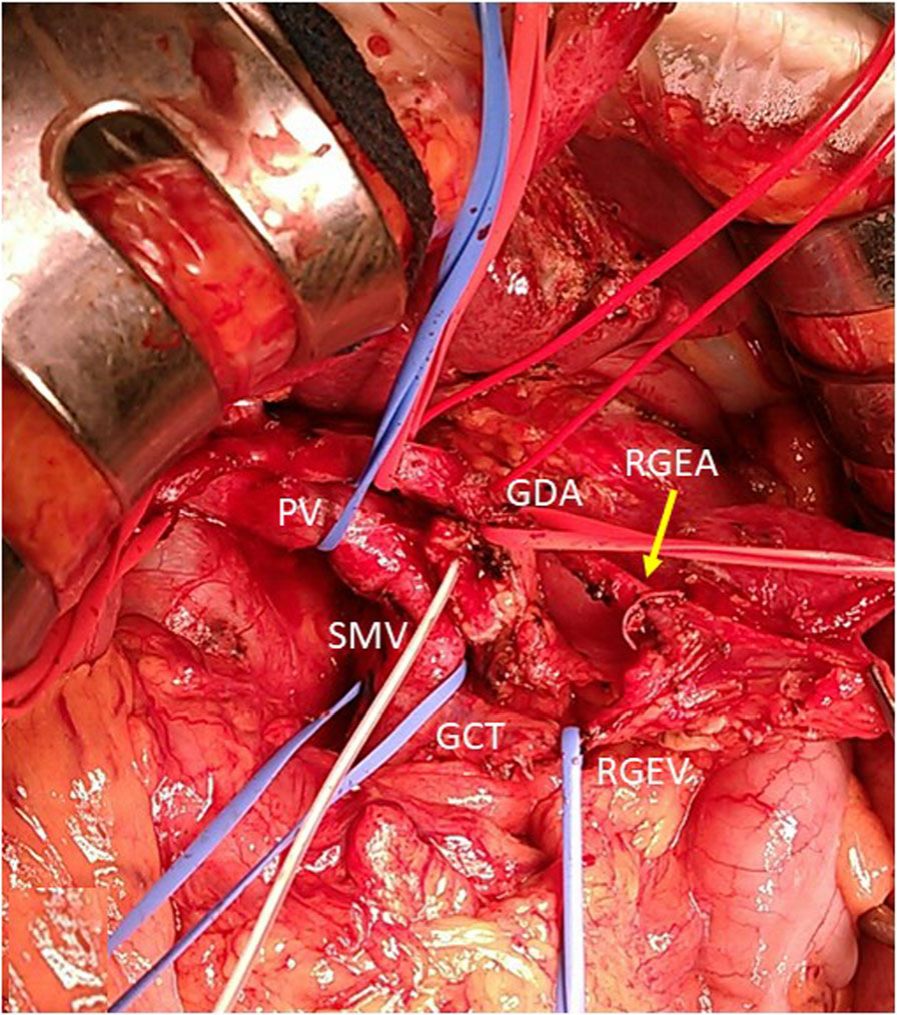
The RGEA was preserved via the GDA, and the RGEV was also preserved via the GCT. RGEA, right gastroepiploic artery; GDA, gastroduodenal artery; RGEV, right gastroepiploic vein; GCT, gastrocolic trunk; PV, portal vein; SMV, superior mesenteric artery

**Fig. 3:**
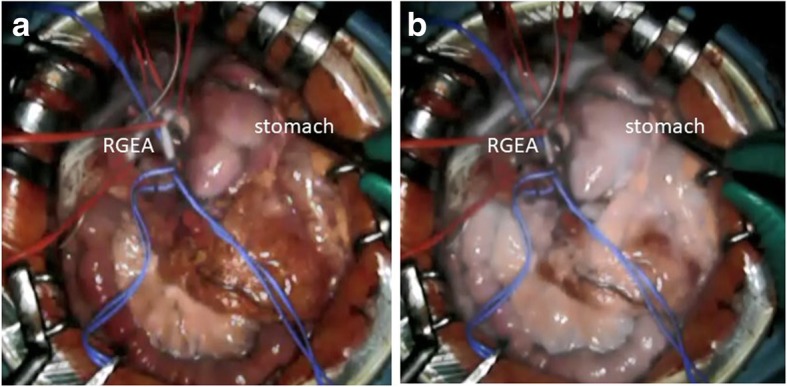
ICG fluorescence. **a** The RGEA glowed white immediately after injection of ICG. **b** The whole remnant stomach glowed white 30 s after injection of ICG. ICG, indocyanine green; RGEA, right gastroepiploic artery

A 73-year-old man underwent PG for gastric cancer. Wall thickening of the common bile duct was detected on a follow-up computed tomography (CT) 2 years after the surgery. Serum biochemistry was as follows: aspartate aminotransferase (AST), 18 U/L; alanine aminotransferase (ALT), 15 U/L; total bilirubin (T-bil), 0.9 mg/dL; carcinoembryonic antigen (CEA), 1.0 ng/mL; and cancer antigen 19-9 (CA 19-9), 12.3 U/mL. A CT scan showed enhanced wall thickening of the common bile duct. Lymph node swelling and vascular invasion were not detected. The right gastric artery (RGA) and right gastroepiploic artery (RGEA) were preserved in the prior operation. Endoscopic retrograde cholangiography (ERC) showed stenosis of the common bile duct with a diameter of 15 mm (Fig. 1a). Positron emission tomography (PET)-CT revealed abnormal fludeoxyglucose uptake at the common bile duct. We diagnosed the patient with common bile duct cancer, and PPPD with preserving the right gastroepiploic vessels was planned with reference to CT reconstructing blood vessels (Fig. 1b). During the PPPD procedure, we preserved the RGEA via the gastroduodenal artery (GDA) and right gastroepiploic vein (RGEV) via the gastrocolic trunk (Fig. 2). We needed to determine whether the remnant stomach could be safely preserved; therefore, an indocyanine green (ICG) fluorescence test was performed (Fig. 3). The results from this test confirmed a good blood supply for the remnant stomach. Pathological examination showed bile duct cancer and pathological stage T2N1M0 stage IIb (TNM classification). The postoperative course was uneventful, and the patient was discharged on postoperative day 29.

### Case 2

**Fig. 4:**
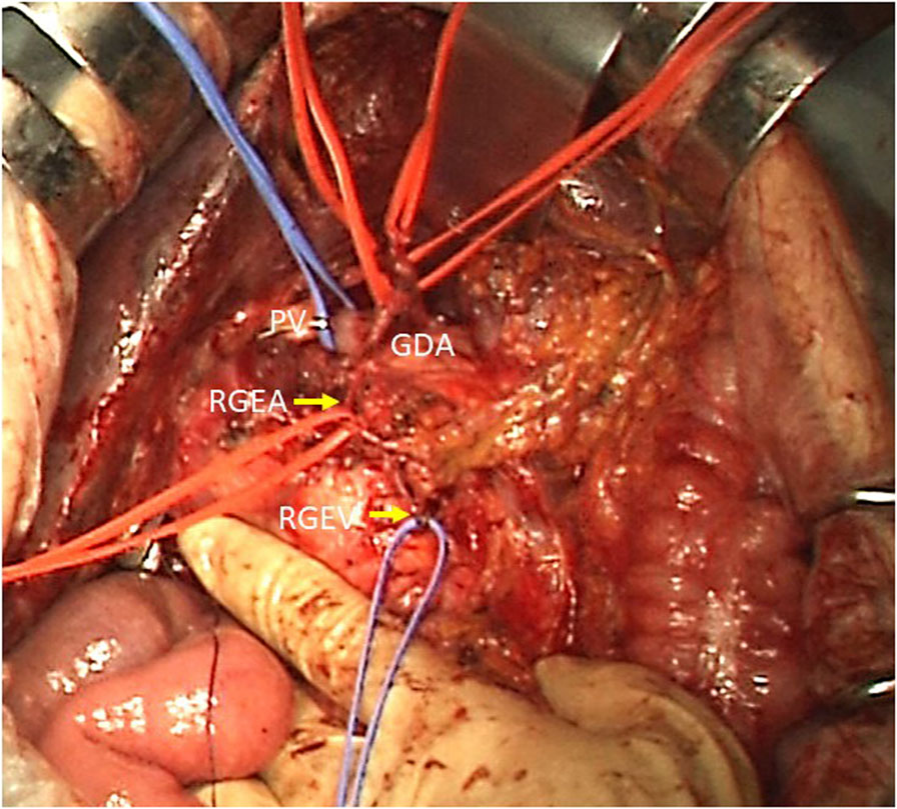
The RGEA was preserved via the GDA, and the RGEV was also preserved. RGEA, right gastroepiploic artery; GDA, gastroduodenal artery; RGEV, right gastroepiploic vein; PV, portal vein

A 58-year-old man was initially admitted to a nearby hospital due to jaundice and detected stenosis of the common bile duct. He was referred to our hospital for further examination. He had histories of PG for gastric cancer 20 years ago and laparoscopic cholecystectomy for cholecystolithiasis 2 years ago. Serum biochemistry was as follows: AST, 27 U/L; ALT, 24 U/L; T-bil, 0.4 mg/dL; CEA, 3.5 ng/mL; and CA 19-9, 80.3 U/mL. A CT scan showed wall thickening of the common bile duct, but lymph node swelling and vascular invasion were not detected. We diagnosed the patient with common bile duct cancer, and PPPD with preserving the right gastroepiploic vessels was planned. During the PPPD procedure, we preserved the RGEA via the GDA and the RGEV via the gastrocolic trunk (Fig. 4) and confirmed a good blood supply for the remnant stomach. Pathological examination showed bile duct cancer and pathological stage T2N1M0 stage IIb (TNM classification). The postoperative course was uneventful, and the patient was discharged on postoperative day 18.

## Discussion

We performed PPPD preserving the right gastroepiploic vessels following PG in two patients. The blood supply of the stomach after PG is maintained in the right gastric and gastroepiploic vessels. When these vessels cannot be preserved, reconstruction of one of these vessels or total resection of the remnant stomach may be necessary in PPPD after PG. Akabane et al. [4] reported the reconstruction of the right gastroepiploic vessels; however, this is a complicated procedure. Ikeda et al. reported PD after esophageal and gastric surgery preserving the right gastroepiploic vessels [5]. If there is no tumor invasion to the GDA, gastrocolic trunk, or right gastroepiploic vessels, it is possible to preserve the remnant stomach.

Pancreatectomy following gastrectomy should be performed with care regarding the blood supply for the remnant stomach [2, 6]. In the distal pancreatectomy (DP) following distal gastrectomy, the remnant stomach cannot be preserved when the blood supply is insufficient. When the left gastric artery was preserved by performing DG for gastric or duodenal ulcers, the remnant stomach could be preserved safely. Even if the left gastric artery was resected by performing DG for gastric cancer, the remnant stomach may be preserved if the blood supply for the stomach from the inferior diaphragm artery or descending branches of the esophageal artery is confirmed [2]. Takahashi et al. [2] reported that two of ten patients who underwent distal pancreatectomy after distal gastrectomy developed severe ischemic complications. Because there were some cases in which the remnant stomach could be preserved, intraoperative evaluation of the blood supply is necessary for preserving the remnant stomach.

It is desirable to confirm the blood supply for the remnant stomach, even if one of the gastric vessels can be preserved or reconstructed in PPPD after PG. Recently, several studies have reported intraoperative assessments of blood supply for the digestive tract [7, 8]. Doppler ultrasonography was trialed for the assessment of vascularization of the intestinal edges during colorectal anastomosis [9]. Akabane et al. reported that near-infrared spectroscopy with in vivo optical spectroscopy (INVOS) which allows real-time monitoring of regional saturation of oxygen was useful for confirming the blood supply for intestinal surgery, and it provided an objective and quantitative assessment of intestinal viability [4]. Recently, ICG fluorescence has been used for the assessment of blood flow for the digestive tract, detection of the liver tumor, cholangiography, and sentinel lymph node mapping [10–12]. We have shown that ICG fluorescence can be used to assess the viability of the remnant stomach and is potentially useful for evaluating blood flow to the remnant stomach. If the intraoperative objective measurement of the viability of the remnant stomach is established, the remnant stomach can be preserved more safely in patients who undergo pancreatectomy after gastrectomy.

We preserved the right gastroepiploic vessels via GDA and gastrocolic trunk. The right gastric vessels can be preserved technically; however, we considered that it is easier to perform reconstruction, such as pancreatojejunostomy and duodenojejunostomy, and lymph node dissection of the hepatobiliary ligament by preserving the right gastroepiploic vessels. There are some problems with the preservation of GDA, such as the difficulty of this procedure, lymph node dissection, and intraoperative bleeding. Because the tumors were not close to GDA in our cases, we could preserve RGEA via GDA and perform lymph node dissection as usual. We separated RGEA and GDA from the pancreas and resected the anterior superior pancreatoduodenal artery before cutting the drainage veins including posterior superior pancreatoduodenal vein. Therefore, this procedure did not increase the amount of bleeding. When the tumor is close to GDA, like pancreatic cancer with infiltration to the ventral side, preserving GDA may be difficult in terms of the difficulty of technique and lymph node dissection. It is important to preserve the remnant stomach; however, the reconstruction of these vessels is a complicated procedure and it is uncertain to maintain blood flow with reconstruction of the thin blood vessel. Therefore, we planned to perform residual gastrectomy if the RGEA and/or RGEV could not be preserved in our two cases.

In conclusion, the remnant stomach could be preserved in performing PPPD following PG by preserving the right gastroepiploic vessels. PPPD after PG is not a frequent situation, but it is sometimes necessary. ICG fluorescence is one of the useful intraoperative assessments for evaluating blood flow to the remnant stomach.

## Abbreviations

ALT: Alanine aminotransferase
AST: Aspartate aminotransferase
CA 19-9: Cancer antigen 19-9
CEA: Carcinoembryonic antigen
CT: Computed tomography
ERC: Endoscopic retrograde cholangiography
GDA: Gastroduodenal artery
ICG: Indocyanine green
PET: Positron emission tomography
PG: Proximal gastrectomy
PPPD: Pylorus-preserving pancreatoduodenectomy
RGA: Right gastric artery
RGEA: Right gastroepiploic artery
RGEV: Right gastroepiploic vein
T-bil: Total bilirubin
